# Rituximab Downregulates Gene Expression Associated with Cell Proliferation, Survival, and Proteolysis in the Peripheral Blood from Rheumatoid Arthritis Patients: A Link between High Baseline Autophagy-Related ULK1 Expression and Improved Pain Control

**DOI:** 10.1155/2016/4963950

**Published:** 2016-01-24

**Authors:** Elena V. Tchetina, Anastasya N. Pivanova, Galina A. Markova, Galina V. Lukina, Elena N. Aleksandrova, Andrey P. Aleksankin, Sergey A. Makarov, Aleksandr N. Kuzin

**Affiliations:** ^1^Clinical Immunology Department, Nasonova Research Institute of Rheumatology, Moscow 115522, Russia; ^2^Clinical Pharmacology Department, Nasonova Research Institute of Rheumatology, Moscow 115522, Russia; ^3^Department of Surgery, Nasonova Research Institute of Rheumatology, Moscow 115522, Russia; ^4^Forensic Medicine Service, Moscow City Health Department, Moscow 115516, Russia

## Abstract

*Objective.* To clarify molecular mechanisms for the response to rituximab in a longitudinal study.* Methods.* Peripheral blood from 16 RA patients treated with rituximab for a single treatment course and 26 healthy controls, blood and knee articular cartilages from 18 patients with long-standing RA, and cartilages from 14 healthy subjects were examined. Clinical response was assessed using ESR, ACPA, CRP, RF, DAS28 levels, CD19+ B-cell counts, bone erosion, and joint space narrowing scores. Protein expression in PBMCs was quantified using ELISA. Gene expression was performed with quantitative real-time PCR.* Results.* A decrease (*p* < 0.05) in DAS28, ESR, and CRP values after rituximab treatment was associated with the downregulation of MTOR, p21, caspase 3, ULK1, TNF*α*, IL-1*β*, and cathepsin K gene expression in the peripheral blood to levels found in healthy subjects. MMP-9 expression remained significantly higher compared to controls although decreased (*p* < 0.05) versus baseline. A negative correlation between baseline ULK1 gene expression and the number of tender joints at the end of follow-up was observed.* Conclusions.* The response to rituximab was associated with decreased MTOR, p21, caspase 3, ULK1, TNF*α*, IL-1*β*, and cathepsin K gene expression compared to healthy subjects. Residual increased expression in MMP-9, IFN*α*, and COX2 might account for remaining inflammation and pain. High baseline ULK1 gene expression indicates a good response in respect to pain.

## 1. Introduction

Rheumatoid arthritis (RA) is one of the most common autoimmune rheumatic diseases and represents a complex systemic multifactorial inflammatory process involving joint destruction [1, 2]. The synovium is a major site of the pathogenesis because activated fibroblast-like synoviocytes produce prostanoids, cytokines, chemokines, matrix degrading enzymes, angiogenic factors, and adhesion molecules [3]. In addition, synovial tissue is invaded by macrophages and activated lymphocytes. T-lymphocytes are involved in the production of a wide range of proinflammatory cytokines, predominantly from the tumor necrosis factor and interleukin superfamilies, and growth factors. B-lymphocytes are associated with the production of autoantibodies, such as rheumatoid factor (RF) and anticyclic citrullinated peptide antibodies (ACPA) [4].

Rituximab (RTX) is a chimeric monoclonal mouse or human antibody IgG1k binding surface antigen, CD20 [5–10], which is expressed in B-cells at different differentiation stages excluding B-cell precursors or terminally differentiated plasma cells [11]. After binding, RTX induces depletion of B-cells by complement- or antibody-mediated cellular cytotoxicity, although some noncirculating tissue B-cells could bind to RTX without causing depletion [12, 13]. RTX in combination with methotrexate (MTX) has been shown to decrease RA disease activity [14–16] and slow joint destruction [17], whereas the latter often occurs independent of patient clinical response [17].

The cellular mechanisms for RTX activity in RA are not clear at present. Several studies have sought to identify RTX response genes based on baseline gene expression signatures in peripheral blood from RA patients. These studies have shown that RTX responders exhibited low expression of genes related to the interferon pathway [18, 19] and arginase (Arg1) as well as low levels of chemokines carrying the C-X-C sequence (CXCL13), a B-cell chemoattractant [20]. Upregulation of genes associated with macrophage and T-cell function, tumor necrosis factor (TNF) receptor-associated factor (TRAF) 1, toll-like receptor (TLR) 4 [21], nuclear factor (NF) kB-related genes, including interleukin- (IL-) 33, and signal transducer and activator of transcription (STAT) 5A [19] was also found in the responders. In contrast, high expression of interferon- (IFN-) *α* [22, 23] and genes related to tissue remodeling, namely, clusterin, tissue inhibitor of metalloproteinase (TIMP) 3, and metalloproteinase (MMP) 9 in synovial tissue [24], was observed in the nonresponders.

A longitudinal study of synovial tissue showed downregulation of genes, encoding immunoglobulins, chemotaxis, leucocyte activation, and immune response after RTX therapy, whereas gene expression associated with cell developmental processes and tissue regeneration increased [25]. Another longitudinal study has shown that good responders demonstrated increased expression of type I IFN-response genes [18]. However, no comparison of RA patient gene expression changes after RTX treatment with gene expression in healthy subjects has been performed.

Our previous studies have shown that methotrexate (MTX) treatment for RA patients significantly decreased the gene expression of the proinflammatory cytokine tumor necrosis factor (TNF) *α* to a level found in healthy controls after 24 months of follow-up [26]. However, the initially increased expression of the non-tissue specific regulatory genes, MTOR (mechanistic target of rapamycin), the major regulator of cell growth and proliferation; ULK1, an autophagy marker involved in longevity and autonomous cell survival; p21, a cyclin-dependent kinase inhibitor; and caspase 3, an apoptosis indicator, was not significantly affected by this drug. Gene expression of the proteases cathepsin K and MMP-9, which are involved in bone and articular cartilage degradation, was also upregulated in the peripheral blood of RA patients compared to healthy subjects and demonstrated variable changes through the course of MTX treatment. Further upregulation of MMP-9 and cathepsin K gene expression compared to baseline in seropositive RA patients treated with MTX was associated with an increase in erosion numbers. Seronegative RA patients exhibited an absence of significant changes in MMP-9 and cathepsin K gene expression in the blood and no increase in the erosion score at the end of the study [26].

To further investigate the mechanisms of antirheumatic drug actions, we analyzed changes in clinical, immunological, and radiological indices and gene expression in the above non-tissue specific regulatory genes, proteases, and cytokines TNF*α*, IL-1*β*, COX2, and IFN*α* in peripheral blood from RA patients before and after one cycle of RTX therapy compared to healthy subjects. We hypothesized that the ability of RTX to ameliorate RA manifestations and delay joint degradation in responsive subjects might be associated with the downregulation of genes involved in bone and articular cartilage destruction and non-tissue specific regulatory genes in the peripheral blood. We found that a significant decrease in DAS28, ESR, and CRP values after RTX therapy was associated with downregulation of MTOR, caspase 3, ULK1, TNF*α*, IL-1*β*, and cathepsin K gene expression to levels found in healthy subjects.

## 2. Patients and Methods

### 2.1. Ethics Statement

Our study was in compliance with the Helsinki Declaration. The study protocol was approved by the Local Committee on the Ethics of Human Research, and informed consent was obtained from all subjects.

### 2.2. Patients

Inclusion criteria of the control subjects: the control group consisted of 26 subjects, (average age 51.1 ± 13.2 years, range 19–69 years) with no current chronic or acute infection and no family history of autoimmune diseases.

Inclusion criteria for the RA patients: the RA patient group consisted of 16 consecutive, unrelated rheumatoid arthritis patients (average age 53.4 ± 10.8 years, range 18–68 years), who visited the clinic at the Nasonova Institute of Rheumatology. The inclusion criteria involved a diagnosis of RA, as defined by the American College of Rheumatology (ACR) 2010 [27], and age ≥18 years (with previous failure of DMARD and anti-TNF*α* blockers).

The exclusion criteria were manifestations of any microbial or viral infections less than two weeks before screening, any symptoms of not entirely controlled systemic, neurological, or psychiatric disease, any malignant or premalignant manifestations at present or in the last five years, and women who were pregnant or lactating.

All patients included in this study received one cycle of RTX therapy at one of two different doses: eight patients received 0.5 g and another eight patients received 1 g. In addition, seven out of 16 patients received MTX (15–20 mg per week), two patients were treated with leflunomide (20 mg daily), another two patients received hydroxychloroquine (200–400 mg daily), and one was treated with azathioprine (150 mg daily). In addition, 11 patients received methylprednisolone (3–12 mg daily), and 14 patients were also treated with NSAIDs. Each patient was followed up by the same investigator through the entire study period.

Another RA patient group consisted of 18 consecutive, unrelated long-standing RA patients undergoing knee joint replacement surgery (average age 50.6 ± 12.9 years, range 31–65 years) diagnosed with RA, as defined by the American College of Rheumatology (ACR) 2010 [27].

### 2.3. Cartilage

Human femoral condylar cartilage was obtained at total knee arthroplasty from the same long-standing 18 patients (average age 50.6 ± 12.9 years, range 31–65 years) with RA diagnosed according to the criteria of the American College of Rheumatology [27].

Human articular cartilage from 14 healthy individuals (average age 38.5 ± 5.9 years, range 29–51 years) (controls) was obtained less than 12 hours postmortem at autopsy from the femoral condylar surfaces of the knee that articulate with the patella.

### 2.4. Demographic, Clinical, and Immunologic Assessment

The evaluation data were collected at baseline and six months. These data included age, gender, disease duration, Steinbrocker's radiographic stage [28], duration of morning stiffness (min), and the disease activity score (DAS) using a modified index involving 28 joints [27, 29]. Concentrations of serum C-reactive protein (cut-off value, 5 mg/L) and IgM class rheumatoid factor (RF) (a standard cut-off value of 15 mU/L was used) were measured via nephelometry using a BN-100 analyzer (Dade Bering, Germany). Anticitrullinated protein autoantibodies (ACPA) were detected using ELISA according to the manufacturer's recommendations (the cut-off level was set at 5 U/mL for antibody positivity) (Axis Shield Diagnostics Limited, Great Britain).

The percentage of CD19+ B-cells in the peripheral blood was determined by flow cytometry using the monoclonal antibodies Cyto-Stat Tetra Chrome CD45-FITC/CD56-RD-1/CD19-ECD/CD3-PC5 and Cyto-Stat Tetra Chrome CD45-FITC/CD56-RD-1/CD8-ECD/CD3-PC5 (Beckman Coulter, USA) with a Cytomics FC 500 cytometer equipped with Cell Quest software according to the manufacturer's recommendations (Beckman Coulter, USA).

### 2.5. Radiographic Assessment

Radiographs of hands and feet were obtained at months 0 and 12. The radiographs were evaluated blind and in chronological order by two independent observers and scored using Sharp's method as modified by van der Heijde [30, 31]. For each patient, an erosion and joint space narrowing score was registered for hands and feet at baseline and after one year, and the mean of the scores from two observers was used to determine the final radiographic scores for erosions and joint space narrowing.

### 2.6. Peripheral Blood Fractionation

Peripheral blood (10 mL) was collected in Vacutainer tubes containing ethylenediaminetetraacetic acid (EDTA) (BDH, England). The blood samples were taken in a standardized manner in the morning (between 07:00 AM and 09:00 AM). Whole blood fractionation was performed using a Ficoll density gradient [32]. PBMCs were collected and washed twice in phosphate-buffered saline (PBS). The obtained cell fractions were frozen and kept at –70°C prior to protein extraction.

### 2.7. Quantification of p70-S6K, p21, MMP-9, and Caspase 3 Protein Levels

Concentrations of phospho-p70-S6K (KHO0581), p21WAF1/Cip1 (KHO5421), MMP-9 (KHC3061), and active caspase 3 (KHO1091) were determined in isolated PBMCs using commercially available enzyme linked immunosorbent assay (ELISA) kits (Invitrogen, Camarillo, CA, USA) according to the manufacturer's instructions. For MTOR protein expression, we evaluated levels of p70-S6K, an MTOR direct target for phosphorylation, which is usually used as an MTOR readout [33, 34]. One unit of p70-S6K [pT389] standard is equivalent to the amount of p70-S6R [pT389] phosphorylated from 1.0 ng of total p70-S6K protein.

PBMC lysates were obtained using Cell Extraction Buffer containing 10 mM Tris, pH 7.4, 100 mM NaCl, 1 mM EDTA, 1 mM EGTA, 1 mM NaF, 20 mM Na_4_P_2_O_7_, 20 mM Na_3_VO_4_, 1% Triton X-100, 10% glycerol, 0.1% SDS, and 0.5% deoxycholate (Invitrogen, Camarillo, CA, USA) supplemented with Protease Inhibitor Cocktail (Sigma-Aldrich, Inc., St. Louis, USA) and 1 mM PMSF (Sigma-Aldrich, Inc., St. Louis, USA) according to the manufacturer's instructions. Total DNA content in PBMC lysates was measured spectrophotometrically using GeneQuant (Amersham Biosciences, United States). Results were expressed per *μ*g of DNA.

### 2.8. Total RNA Isolation and Reverse Transcriptase (RT) Reaction

For detection of gene expression total RNA was isolated from 100 *μ*L of whole blood immediately after withdrawal using Ribo-zol-A kit (InterLabService, Moscow, Russia) in accordance with the manufacturer's recommendations. Total RNA was also isolated from fresh knee articular cartilage using TRIzol reagent according to the manufacturer's recommendations (Invitrogen, Carlsbad, CA USA). Total RNA had *A*_260/290_ > 1.9. The RT reaction was performed using a Reverta kit containing M-MLV Reverse Transcriptase, random hexanucleotide primers, and total RNA according to the manufacturer's recommendations (InterLabService, Moscow, Russia).

### 2.9. Real-Time Quantitative PCR

The following premade primers and probes were used for the TaqMan assay (Applied Biosystems, Foster City, CA, USA): MTOR (Hs00234522_m1), Unc-51-like kinase 1 (ULK1) (Hs00177504_m1), p21WAF1/Cip1 (p21) (Hs00355782_m1), caspase 3 (Hs00263337_m1), TNF*α* (Hs00174128_m1), IL-1*β* (Hs00174097_m1), COX2 (Hs00153133-m1), IFN*α* (Hs00855471_m1), MMP-9 (Hs00234579_m1), and cathepsin K (Hs00166165_m1). *β*-Actin was used as an endogenous control.

The quantification of gene expression was conducted using a 7300 Real-Time PCR System (Applied Biosystems, Foster City, CA, USA) as described previously [35]. Briefly, 1 *μ*L of RT product was subjected to real-time PCR in a 15 *μ*L total reaction mixture containing 7.5 *μ*L of TaqMan Universal PCR Master Mix (Applied Biosystems), 900 nM sense and antisense primers, 50 nM probe, and template cDNA. After a single step of 50°C for 2 min and an initial activation at 95°C for 10 min, the reaction mixtures were subjected to 40 amplification cycles (15 s at 95°C for denaturation and 1 min of annealing and extension at 60°C).

Relative mRNA expression was determined using the delta-delta C_T_ method, as detailed by the manufacturer guidelines (Applied Biosystems) [36]. The delta C_T_ value was calculated by subtracting the C_T_ value for the housekeeping *β*-actin gene from the C_T_ value for each sample. A delta-delta C_T_ value was then calculated by subtracting the delta C_T_ value of the control (each healthy patient) from the delta C_T_ value of each RA patient. Each PCR was performed in duplicate. Three “no template” controls were consistently negative for each reaction.

### 2.10. Statistical Analysis

The descriptive values of the variables, which did not have a Gaussian distribution, were expressed as medians and interquartile ranges. A statistical comparison between the independent patient groups was performed using the Mann-Whitney *U* test and Spearman's rank correlations. For the statistical comparison between the RA patient groups before and after treatment, the Wilcoxon matched pairs test was applied. To compare percentages, a one-tailed *Z*-test for percentages was applied. Statistica 6 software (StatSoft, Tulsa, OK, USA) was used for all of the statistical analyses. *p* values ≤ 0.05 were considered to be significant.

## 3. Results

### 3.1. Whole Blood Gene Expression in Rheumatoid Arthritis Patients at Baseline and at Six Months

**Figure 1:**
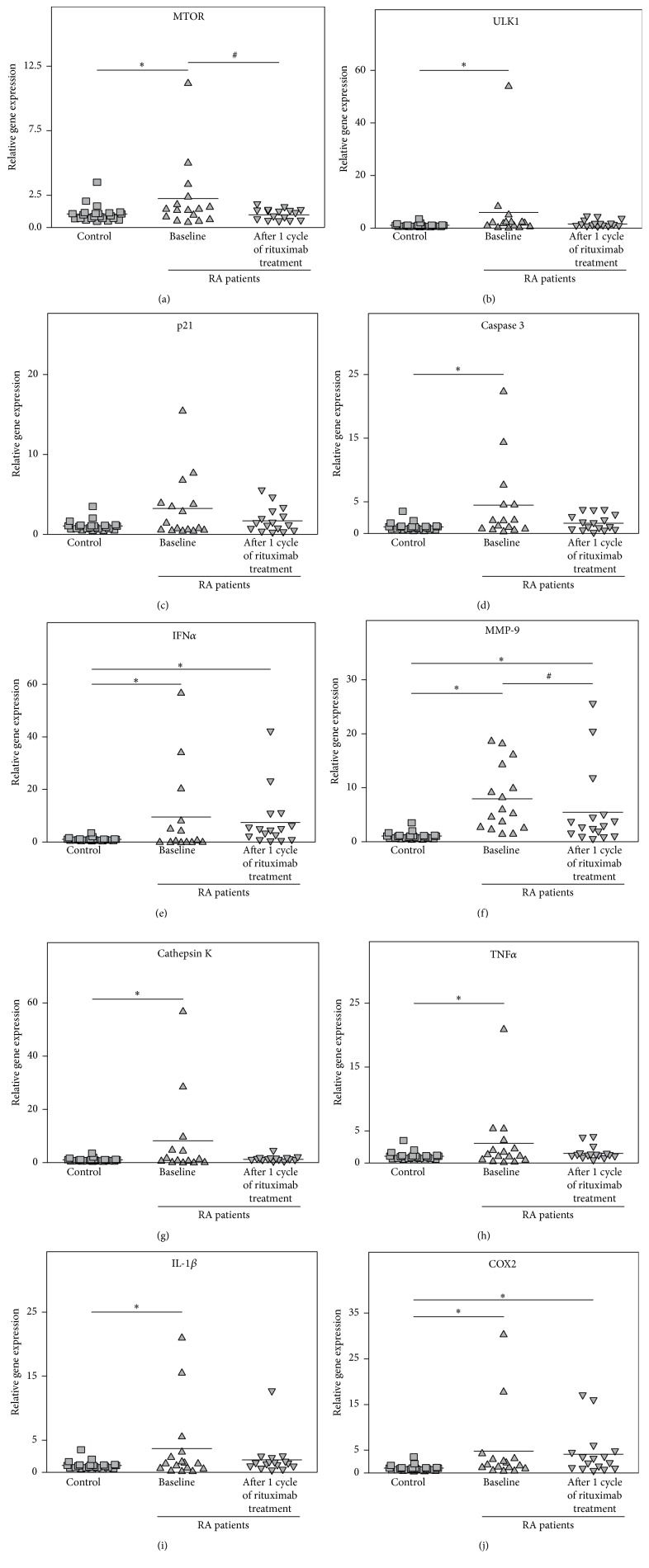
Relative expression of the genes MTOR (a), ULK1 (b), p21 (c), caspase 3 (d), IFN*α* (e), MMP-9 (f), cathepsin K (g), TNF*α* (h), IL-1*β* (i), and COX2 (j) with reference to *β*-actin as determined using real-time PCR analyses in whole blood from RA patients (*n* = 16) compared to healthy controls (*n* = 26). The controls are shown as 1.0 as required for relative quantification with the real-time PCR protocol. Asterisks (*∗*) indicate significant differences from the control in pairwise comparisons (Mann-Whitney *U* test). The number signs (#) show significant differences from baseline in pairwise comparisons (Wilcoxon matched pairs test).

The majority of the examined genes, including the regulator of cell growth and proliferation MTOR; the autophagy marker ULK1; the apoptosis indicator caspase-3; the proinflammatory cytokines TNF*α*, IL-1*β*, IFN*α*, and COX2; and the proteases MMP-9 and cathepsin K, were significantly upregulated in a sample of RA patients (*n* = 16) at baseline compared to healthy subjects (Figure 1). Although there was a considerable variation in ULK1 gene expression, plotting the data without an outlier confirmed that RA patients exhibited significantly upregulated ULK1 gene expression (*p* = 0.01) at baseline (Supplementary Material, Figure  4, available online at http://dx.doi.org/10.1155/2016/4963950). RTX treatment resulted in a significant decrease in MTOR (*p* = 0.05) and MMP-9 (*p* = 0.01) gene expression compared to baseline. Expression of the majority of examined genes also decreased and became comparable to those of healthy controls (*p* > 0.05), whereas expression of MMP-9, COX-2, and IFN*α* remained significantly higher than in control subjects (*p* < 0.01). Therefore, RTX therapy was effective in downregulating genes related to growth, proliferation, cell survival, apoptosis, and proinflammatory cytokines TNF*α* and IL-1*β* and partially effective for genes related to bone tissue destruction. However, expression of the metalloproteinase MMP-9 and proinflammatory mediators COX2 and IFN*α* remained high.

An analysis of bivariate correlations using Spearman's correlation coefficient for the expression of MTOR, ULK1, p21, caspase 3, and TNF*α* at baseline showed positive correlations (*p* < 0.05) with each other in the examined RA patients (*n* = 16) (Table 1). Cathepsin K gene expression positively correlated with only MMP-9 and TNF*α*. IL-1*β* gene expression correlated with only COX2 and IFN*α*. A positive correlation was also noted between p21, cathepsin K, and COX2 gene expression and serum RF protein level.

An assessment with Spearman's correlation coefficient for the expression of the examined genes at baseline versus clinical and immunological indices measured after six months of follow-up showed a positive correlation (*p* ≤ 0.05) of ULK1, caspase 3, and MMP-9 gene expression with serum ACPA protein levels as well as p21 and cathepsin K gene expression with serum RF levels (Table 2). However, the most important observation was related to a negative correlation between ULK1 baseline gene expression and the number of tender joints measured at the end of the study (Table 2). In contrast, baseline ULK1 gene expression versus that at the end of follow-up in the same RA patients revealed a positive correlation (Spearman's correlation coefficient *r* = 0.731, *p* < 0.05).

**Figure 2:**
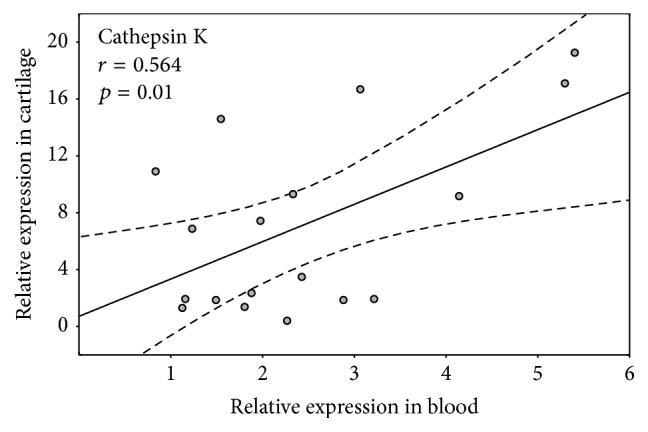
A relationship between cathepsin K gene expression measured in the blood or articular cartilage from long-standing RA patients (*n* = 18).

Plotting cathepsin K and MMP-9 gene expression in PBMCs versus articular cartilage from the same 18 patients with long-standing RA revealed a significant correlation (Pearson's correlation coefficient *r* = 0.564; *p* = 0.01) for only cathepsin K (Figure 2).

### 3.2. Protein Expression of Phospho-p70-S6K, p21, MMP-9, and Active Caspase 3 in Isolated PBMCs

**Figure 3:**
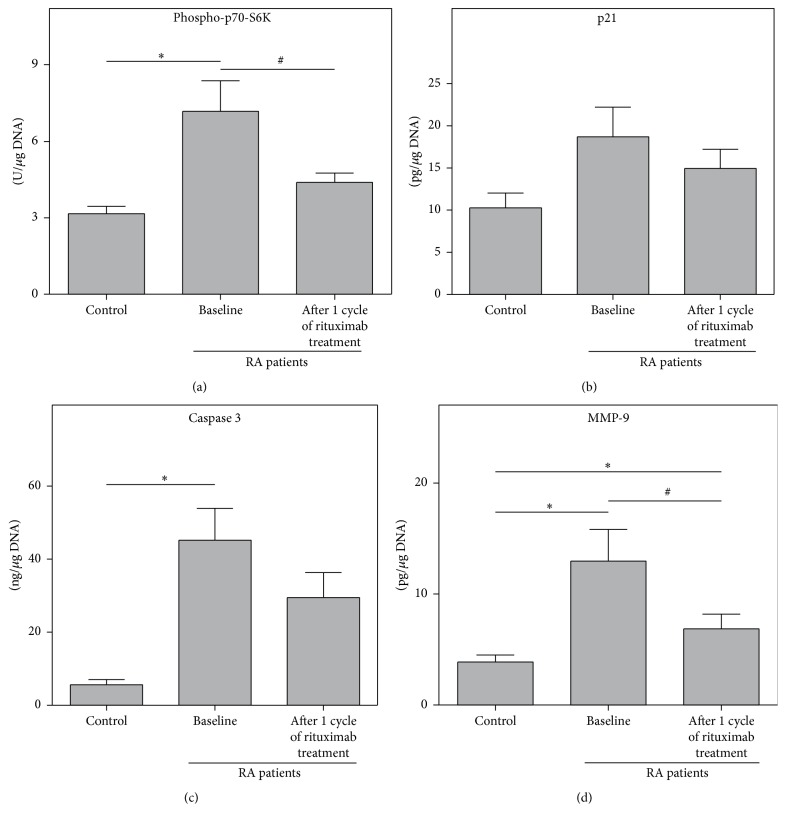
Protein concentrations of phospho-p70-S6K (a), p21 (b), active caspase 3 (c), and MMP-9 (d) measured using ELISA in PBMCs from RA patients (*n* = 16) compared to controls (*n* = 26). Asterisks (*∗*) indicate significant differences from the healthy control patients in pairwise comparisons (Mann-Whitney *U* test). The number signs (#) show significant differences from baseline in pairwise comparisons (Wilcoxon matched pairs test).

To further investigate the clinical significance of MTOR, p21, MMP-9, and caspase 3 relative gene expression in whole blood from RA patients we analyzed the protein levels of phospho-p70-S6K serine/threonine kinase (a direct target for phosphorylation by mTOR [33, 34]), p21, MMP-9, and active caspase 3 in the PBMC fraction. These studies showed that at baseline phospho-p70-S6K, MMP-9, and caspase 3 protein concentrations in PBMCs were significantly higher in RA patients than in healthy controls (Figure 3). At the end of the study, protein expression of phospho-p70-S6K, p21, and caspase 3 was close to control values, whereas the MMP-9 concentration though significantly decreased compared to the baseline remained much higher than those of healthy subjects.

### 3.3. Clinical, Immunological, and Radiological Parameters at Baseline and Six Months and the Therapeutic Response to RTX in Rheumatoid Arthritis Patients

The mean disease duration at enrollment was 8.2 ± 7.1 years. Seven patients had Steinbrocker's radiographic stage II; another seven had stage III; and two patients had stage IV both at baseline and after six months of follow-up (Table 3). All of the patients were ACPA-positive, whereas 15 of 16 were RF positive at baseline. The average ACPA and RF levels did not change significantly through the course of treatment. RF IgM levels reached normal values in only two of the examined patients by the end of the study. ESR and CRP levels despite a significant decrease (*p* < 0.05) remained high by the end of the study and only eight and four patients, respectively, reached normal values. Most patients (14 out of 16) exhibited high disease activity (DAS28 > 5.1) at study entry. The DAS28 index decreased significantly after six months of follow-up (*p* = 0.0001). An average change in the disease activity score (ΛDAS) was 2.6 (varied from 1.2 to 5.0). All of the patients were responsive to RTX with 1.2 or higher ΛDAS values observed after six months of follow-up. However, only three patients fulfilled the remission criteria (DAS28 < 2.6), and low disease activity (2.6 < DAS28 < 3.2) was observed in five patients. Another five patients exhibited moderate disease activity (3.2 < DAS28 < 5.1), whereas three patients maintained high disease activity at the end of the study.

These findings were associated with significant decreases in morning stiffness and the number of swollen and tender joints at the end of the study. However, swollen joints were observed in 12 of 16 patients, whereas tender joints were found in 13 of 16 patients by the end of follow-up. All of the RA patients demonstrated almost complete depletion of CD19+ B-cells (*p* = 0.0002). The number of bone erosions showed no change, whereas the joint space narrowing score slightly increased after one-year followed treatment compared to baseline values.

## 4. Discussion

Almost all phenotypic aspects of RA are determined by pathophysiological processes encoded by genes and their products, which are responsible for the unique pathogenesis of the disease [37, 38]. Therefore, our longitudinal study on the changes in gene expression in peripheral blood from RA patients during RTX therapy permitted some insights into the mechanisms of drug action at the molecular level.

In particular, the improvement in clinical indices was associated with decreased expression of genes responsible for regulating cell proliferation (MTOR, p21), survival (ULK1), and apoptosis (caspase 3) in the peripheral blood from RA patients after RTX treatment to the level observed in healthy controls. This finding might indicate the recovery of a healthier cellular phenotype in these cells. Downregulation of caspase 3 expression in the examined RA patients to the level of healthy subjects after RTX therapy contradicts the notion that stimulation of apoptosis could be one of the mechanisms for RTX-mediated B-cell elimination [39] and supports a belief that the ability of RTX to induce apoptosis is very low [40]. We also noted a difference in the gene expression outcome after RTX treatment with the MTX effects observed in our previous studies where the gene expression of ULK1, p21, and caspase 3 was still upregulated at the end of the follow-up period in blood from RA patients treated with MTX [26].

The significant decrease in MMP-9 gene expression compared to baseline and downregulation of cathepsin K expression to healthy control values were accompanied by the absence of any increase in the erosion number and a negligible increase in joint space narrowing one year after RTX treatment. Because these genes are responsible for bone and articular cartilage destruction in RA [41], our results support previous observations for the ability of RTX to slow joint degeneration [17]. In contrast, after MTX therapy for RA patients, MMP-9 and cathepsin K gene expression remained upregulated, and this finding was associated with persistent joint degrading activity [26]. Moreover, a positive correlation of cathepsin K gene expression in the peripheral blood and articular cartilage of the same patients with long-standing RA who were undergoing arthroplasty indicates that this gene expression in the blood might mirror its activity in the joint and serve a blood-based marker for joint degradation.

The gene expression changes observed here were accompanied by almost complete B-cell depletion in the blood, which was also noted in other studies [42, 43]. We also observed a significant decrease in disease activity (registered by DAS28), stiffness, number of tender and swollen joints, ESR, and CRP serum levels. Although all of the patients responded to RTX therapy as demonstrated by a decrease in DAS28 ≥ 1.2, their average disease activity decreased only to a moderate level and was accompanied by increased serum levels of RF, ACPA, ESR, and CRP, stiffness, and persistence of several tender and swollen joints.

Retention of the inflammatory process, as manifested by the maintenance of swollen joints in 12 of 16 patients and moderate disease activity, was observed despite a significant decrease in TNF*α* and IL-1*β* gene expression to the level found in healthy controls. The remaining inflammation might be associated with a high residual expression of COX2 and IFN*α*, which were shown to be capable of inducing RA symptoms [44–47]. This finding supports previous observations that RTX can slow joint destruction independent of clinical outcome [17].

In addition, high residual expression of MMP-9 and COX2 could be associated with joint pain maintenance in the majority of the examined RA patients because these gene products were previously found to be responsible for neuropathic pain in animal studies [48, 49].

Although association of autophagy in pain processing is not sufficiently studied at present [50], autophagy might be involved in pain control as its induction by rapamycin, pentobarbital, or morphine was accompanied by long-lasting analgesic effects while its inhibition by chloroquine or miR-195 aggravated neuropathic pain following peripheral nerve injury in animal studies [51–54]. A positive correlation of baseline ULK1 expression with that at the end of the follow-up indicates an improved capacity of RA patients with high baseline ULK1 gene expression to maintain sufficient levels of autophagy activity for pain regulation. This is supported by the observation that stimulation of autophagy was associated with suppression of clinical arthritis and inflammatory cytokine production in RA [55, 56]. Therefore, a negative correlation observed here for baseline expression of autophagy-related ULK1 gene with the number of tender joints at the end of RTX therapy suggests that RA patients with high expression of this gene might better respond to RTX in terms of pain control. Therefore, controlling ULK1 expression before RTX therapy could be reasonable.

In conclusion, our study shows that after one cycle of RTX therapy the improvement in clinical and laboratory indices in RA patients was associated with the downregulation of the non-tissue specific regulatory genes MTOR, ULK1, p21, caspase 3, cytokines TNF*α* and IL-1*β*, and protease cathepsin K to healthy control levels. The maintenance of increased expression of MMP-9, IFN*α*, and COX2 might be associated with persistent moderate disease activity and maintenance of tender and swollen joints in the majority of RA patients after one cycle of RTX therapy. In addition, a high baseline gene expression of ULK1 might indicate a better response to RTX treatment in terms of pain control. Further studies involving larger patient cohorts are required to confirm our observations and to identify new gene expression biomarkers for personalized prediction of RTX response.

## Supplementary Material

The autophagy marker ULK1 was significantly upregulated in a sample of RA patients (n = 16) at baseline compared to healthy subjects (Figure 1). Although there was a considerable variation in ULK1 gene expression, plotting the data without an outlier (n = 15) confirmed that RA patients exhibited significantly upregulated ULK1 gene expression (p = 0.01) at baseline (Supplementary Material, Figure 4).

## Figures and Tables

**Table 1:** Spearmen's correlation coefficients and their significance (*p*) are shown for the expression of the examined genes in relation to each other and immunological marker in RA patients (*n* = 16) at baseline.

	ULK1	P21	Caspase 3	MMP-9	Cathepsin K	TNF*α*	COX2	IFN*α*
MTOR	0.891 *p* < 0.001	0.607 *p* = 0.01	0.878 *p* < 0.001	0.597 *p* = 0.01	0.859 *p* < 0.001	0.747 *p* < 0.001		

ULK1		0.800 *p* = 0.003	0.963 *p* < 0.001	0.729 *p* = 0.002	0.906 *p* < 0.001	0.792 *p* < 0.001		

P21			0.773 *p* < 0.001	0.600 *p* = 0.01	0.954 *p* < 0.001	0.758 *p* < 0.001		

Caspase 3				0.723 *p* = 0.002	0.927 *p* < 0.001	0.797 *p* < 0.001		

MMP-9					0.651 *p* = 0.01			

Cathepsin K						0.840 *p* < 0.001		

IL-1*β*							0.611 *p* = 0.01	0.608 *p* = 0.002

RF		0.585 *p* = 0.01			0.607 *p* = 0.02		0.529 *p* = 0.03	

**Table 2:** Spearmen's correlation coefficients and their significance (*p*) are shown for the baseline expression of the examined genes in relation to immunological markers at the end of one cycle of RTX treatment of RA patients (*n* = 16).

Clinical and immunological indices after one cycle of RTX therapy (*n* = 16)	ULK1	P21	Caspase 3	MMP-9	Cathepsin K
АСPA	0.736 *p* = 0.004		0.710 *p* = 0.006	0.600 *p* = 0.02	

RF		0.582 *p* = 0.01			0.616 *p* = 0.01

Number of tender joints	−0.514 *p* = 0.04				

**Table 3:** Clinical, immunological, and radiological parameters at baseline and after one cycle of RTX treatment in rheumatoid arthritis patients (*n* = 16).

Patients	At baseline (*n* = 16) Ме [25; 75]	After one cycle of RTX treatment (*n* = 16) Ме [25; 75]	*p* (Wilcoxon matched pairs test)
RF, mU/L (range)	282.5 [143.5; 455] (9.5–1980)	172 [96.5; 538.5] (9.5–541.8)	0.67

ACPA, U/mL (range)	200 [111.6; 332.2] (46.2–3016.1)	328.5 [121.2; 501] (0.1–501)	0.54

CRP, mg/mL (range)	27.1 [11.6; 44.1] (5.9–154)	7.9 [5.6; 20.2] (0.4–38.9)	0.01

ESR, mm (range)	45 [35.5; 62.5] (8–87)	21 [16.5; 36] (5–56)	0.001

DAS 28 (range)	6.3 [5.7; 7.1] (4.2–7.6)	3.5 [2.7; 4.5] (1.3–6.44)	0.0001

Δ DAS 28 (range)		2.6 [1.2; 3.7] (1.2–5.0)	

Stiffness (range)	135 [45; 210] (0–240)	7.5 [0; 35] (0–120)	0.001

Number of swollen joints (range)	7 [2.5; 10.5] (2–15)	1 [0.5; 4] (0–8)	0.001

Number of tender joints (range)	16.0 [10; 23.5] (4–26)	2 [1; 7] (0–28)	0.001

Erosion number (range)	7 [0; 22] (0–24)	6 [0; 22] (0–23)	0.5

Joint space narrowing (range)	65 [19; 91] (8–95)	76 [33; 91] (17–95)	0.25

CD19+ В-cells amounts (%)	10.3 [4.2; 13.5] (3.3–24.8)	0.06 [0; 0.32] (0–0.9)	0.0002

## References

[1] Scher J. U. (2012). B-cell therapies for rheumatoid arthritis. *Bulletin of the NYU Hospital for Joint Diseases*.

[2] Lee D. M., Weinblatt M. E. (2001). Rheumatoid arthritis. *The Lancet*.

[3] Bartok B., Firestein G. S. (2010). Fibroblast-like synoviocytes: key effector cells in rheumatoid arthritis. *Immunological Reviews*.

[4] McInnes I. B., Schett G. (2011). The pathogenesis of rheumatoid arthritis. *The New England Journal of Medicine*.

[5] Reff M. E., Carner K., Chambers K. S. (1994). Depletion of B cells in vivo by a chimeric mouse human monoclonal antibody to CD20. *Blood*.

[6] Tedder T. F., Engel P. (1994). CD20: a regulator of cell-cycle progression of B lymphocytes. *Immunology Today*.

[7] Eisenberg R., Looney R. J. (2005). The therapeutic potential of anti-CD20: what do B-cells do?. *Clinical Immunology*.

[8] Levesque M. C., St Clair E. W. (2008). B cell-directed therapies for autoimmune disease and correlates of disease response and relapse. *The Journal of Allergy and Clinical Immunology*.

[9] Hultin L. E., Hausner M. A., Hultin P. M., Giorgi J. V. (1993). CD20 (Pan-B cell) antigen is expressed at a low level on a subpopulation of human T lymphocytes. *Cytometry*.

[10] Quintanilla-Martinez L., Preffer F., Rubin D., Ferry J. A., Harris N. L. (1994). CD20^+^ T-cell lymphoma: neoplastic transformation of a normal T-cell subset. *American Journal of Clinical Pathology*.

[11] Anderson K. C., Bates M. P., Slaughenhoupt B. L., Pinkus G. S., Schlossman S. F., Nadler L. M. (1984). Expression of human B cell-associated antigens on leukemias and lymphomas: a model of human B cell differentiation. *Blood*.

[12] Martin F., Chan A. C. (2006). B cell immunobiology in disease: evolving concepts from the clinic. *Annual Review of Immunology*.

[13] Di Gaetano N., Cittera E., Nota R. (2003). Complement activation determines the therapeutic activity of rituximab in vivo. *Journal of Immunology*.

[14] Emery P., Fleischmann R., Filipowicz-Sosnowska A. (2006). The efficacy and safety of rituximab in patients with active rheumatoid arthritis despite methotrexate treatment: results of a phase IIB randomized, double-blind, placebo-controlled, dose-ranging trial. *Arthritis & Rheumatism*.

[15] Cohen S. B., Emery P., Greenwald M. W. (2006). Rituximab for rheumatoid arthritis refractory to anti-tumor necrosis factor therapy: results of a multicenter, randomized, double-blind, placebo-controlled, phase III trial evaluating primary efficacy and safety at twenty-four weeks. *Arthritis and Rheumatism*.

[16] Edwards J. C. W., Szczepański L., Szechiński J. (2004). Efficacy of B-cell-targeted therapy with rituximab in patients with rheumatoid arthritis. *The New England Journal of Medicine*.

[17] Keystone E. C., Emery P., Peterfy C. G. (2009). Rituximab inhibits structural joint damage in patients with rheumatoid arthritis with an inadequate response to tumour necrosis factor inhibitor therapies. *Annals of the Rheumatic Diseases*.

[18] Vosslamber S., Raterman H. G., Van Der Pouw Kraan T. C. T. M. (2011). Pharmacological induction of interferon type I activity following treatment with rituximab determines clinical response in rheumatoid arthritis. *Annals of the Rheumatic Diseases*.

[19] Sellam J., Marion-Thore S., Dumont F. (2014). Use of whole-blood transcriptomic profiling to highlight several pathophysiologic pathways associated with response to rituximab in patients with rheumatoid arthritis: data from a randomized, controlled, open-label trial. *Arthritis & Rheumatology*.

[20] Rosengren S., Wei N., Kalunian K. C., Kavanaugh A., Boyle D. L. (2011). CXCL13: a novel biomarker of B-cell return following rituximab treatment and synovitis in patients with rheumatoid arthritis. *Rheumatology*.

[21] Julià A., Barceló M., Erra A., Palacio C., Marsal S. (2009). Identification of candidate genes for rituximab response in rheumatoid arthritis patients by microarray expression profiling in blood cells. *Pharmacogenomics*.

[22] Thurlings R. M., Boumans M., Tekstra J. (2010). Relationship between the type I interferon signature and the response to rituximab in rheumatoid arthritis patients. *Arthritis and Rheumatism*.

[23] Raterman H. G., Vosslamber S., de Ridder S. (2012). The interferon type I signature towards prediction of non-response to rituximab in rheumatoid arthritis patients. *Arthritis Research & Therapy*.

[24] Hogan V. E., Holweg C. T. J., Choy D. F. (2012). Pretreatment synovial transcriptional profile is associated with early and late clinical response in rheumatoid arthritis patients treated with rituximab. *Annals of the Rheumatic Diseases*.

[25] Gutierrez-Roelens I., Galant C., Theate I. (2011). Rituximab treatment induces the expression of genes involved in healing processes in the rheumatoid arthritis synovium. *Arthritis and Rheumatism*.

[26] Tchetina E. V., Demidova N. V., Karateev D. E., Nasonov E. L. (2013). Rheumatoid factor positivity is associated with increased joint destruction and upregulation of matrix metalloproteinase 9 and cathepsin K gene expression in the peripheral blood in rheumatoid arthritic patients treated with methotrexate. *International Journal of Rheumatology*.

[27] Aletaha D., Neogi T., Silman A. J. (2010). 2010 Rheumatoid arthritis classification criteria: an American College of Rheumatology/European League Against Rheumatism collaborative initiative. *Arthritis & Rheumatism*.

[28] Steinbrocker O., Traeger C. H., Batterman R. C. (1949). Therapeutic criteria in rheumatoid arthritis. *The Journal of the American Medical Association*.

[29] Prevoo M. L. L., van Gestel A. M., van 't Hof M. A., van Rijswijk M. H., van de Putte L. B. A., van Riel P. L. C. M. (1996). Remission in a prospective study of patients with rheumatoid arthritis. American rheumatism association preliminary remission criteria in relation to the disease activity score. *British Journal of Rheumatology*.

[30] van der Heijde D. M. (1999). How to read radiographs according to the Sharp/van der Heijde method. *The Journal of Rheumatology*.

[31] van der Heijde D. M., Dankert T., Nieman F., Rau R., Boers M. (1999). Reliability and sensitivity to change of a simplification of the Sharp/van der Heijde radiological assessment in rheumatoid arthritis. *Rheumatology*.

[32] Son B. K., Roberts R. L., Ank B. J., Stiehm E. R. (1996). Effects of anticoagulant, serum, and temperature on the natural killer activity of human peripheral blood mononuclear cells stored overnight. *Clinical and Diagnostic Laboratory Immunology*.

[33] Isotani S., Hara K., Tokunaga C., Inoue H., Avruch J., Yonezawa K. (1999). Immunopurified mammalian target of rapamycin phosphorylates and activates p70 S6 kinase *α* in vitro. *The Journal of Biological Chemistry*.

[34] Fingar D. C., Salama S., Tsou C., Harlow E., Blenis J. (2002). Mammalian cell size is controlled by mTOR and its downstream targets S6K1 and 4EBP1/eIF4E. *Genes and Development*.

[35] Tchetina E. V., Poole A. R., Zaitseva E. M. (2013). Differences in mTOR (mammalian target of rapamycin) gene expression in the peripheral blood and articular cartilages of osteoarthritic patients and disease activity. *Arthritis*.

[36] Livak K. J. Comparative Ct method, User Bulletin no. 2: ABI PRISM7700 Sequence Detection System, PE Applied Biosystems.

[37] Verweij C. L., Vosslamber S. (2011). New insight in the mechanism of action of rituximab: the interferon signature towards personalized medicine. *Discovery Medicine*.

[38] Tchetina E. V. (2015). ‘High’ and ‘low’ gene expression signatures in rheumatoid arthritis: an emerging approach for patient stratification and therapy choice. *International Journal of Orthopaedics*.

[39] Maloney D. G., Smith B., Appelbaum F. R. (1996). The anti-tumor effect of monoclonal anti-CD20 antibody (mAb) therapy includes direct antiproliferative activity and induction of apoptosis in CD20 positive non-Hodgkin's lymphoma (NHL) cell lines. *Blood*.

[40] Van Meerten T., van Rijn R. S., Hol S., Hagenbeek A., Ebeling S. B. (2006). Complement-induced cell death by rituximab depends on CD20 expression level and acts complementary to antibody-dependent cellular cytotoxicity. *Clinical Cancer Research*.

[41] Troen B. R. (2004). The role of cathepsin K in normal bone resorption. *Drug News and Perspectives*.

[42] Dass S., Rawstron A. C., Vital E. M., Henshaw K., McGonagle D., Emery P. (2008). Highly sensitive B cell analysis predicts response to rituximab therapy in rheumatoid arthritis. *Arthritis and Rheumatism*.

[43] Thurlings R. M., Vos K., Gerlag D. M., Tak P. P. (2008). Disease activity-guided rituximab therapy in rheumatoid arthritis: the effects of re-treatment in initial nonresponders versus initial responders. *Arthritis & Rheumatism*.

[44] Conrad B. (2003). Potential mechanisms of interferon-*α* induced autoimmunity. *Autoimmunity*.

[45] Izumi Y., Komori A., Yasunaga Y. (2011). Rheumatoid arthritis following a treatment with IFN-*α*/ribavirin against HCV infection. *Internal Medicine*.

[46] Yoon H.-Y., Lee E.-G., Lee H. (2013). Kaempferol inhibits IL-1*β*-induced proliferation of rheumatoid arthritis synovial fibroblasts and the production of COX-2, PGE2 and MMPs. *International Journal of Molecular Medicine*.

[47] Cutolo M., Capellino S., Montagna P., Sulli A., Seriolo B., Villaggio B. (2006). Anti-inflammatory effects of leflunomide in combination with methotrexate on co-culture of T lymphocytes and synovial macrophages from rheumatoid arthritis patients. *Annals of the Rheumatic Diseases*.

[48] Kawasaki Y., Xu Z.-Z., Wang X. (2008). Distinct roles of matrix metalloproteases in the early- and late-phase development of neuropathic pain. *Nature Medicine*.

[49] Vardeh D., Wang D., Costigan M. (2009). COX2 in CNS neural cells mediates mechanical inflammatory pain hypersensitivity in mice. *The Journal of Clinical Investigation*.

[50] Berliocchi L., Maiarù M., Varano G. P. (2015). Spinal autophagy is differently modulated in distinct mouse models of neuropathic pain. *Molecular Pain*.

[51] Marinelli S., Nazio F., Tinari A. (2014). Schwann cell autophagy counteracts the onset and chronification of neuropathic pain. *Pain*.

[52] Zhao L., Zhu Y., Wang D. (2010). Morphine induces Beclin 1- and ATG5-dependent autophagy in human neuroblastoma SH-SY5Y cells and in the rat hippocampus. *Autophagy*.

[53] Kashiwagi A., Hosokawa S., Maeyama Y. (2015). Anesthesia with disuse leads to autophagy up-regulation in the skeletal muscle. *Anesthesiology*.

[54] Shi G., Shi J., Liu K. (2013). Increased miR-195 aggravates neuropathic pain by inhibiting autophagy following peripheral nerve injury. *Glia*.

[55] Yan H., Zhou H. F., Hu Y., Pham C. T. (2015). Suppression of experimental arthritis through AMP-activated protein kinase activation and autophagy modulation. *Journal of Rheumatic Diseases and Treatment*.

[56] Michalsen A., Li C. (2013). Fasting therapy for treating and preventing disease—current state of evidence. *Forschende Komplementärmedizin*.

