# Supplementary material for: Rituximab Downregulates Gene Expression Associated with Cell Proliferation, Survival, and Proteolysis in the Peripheral Blood from Rheumatoid Arthritis Patients: A Link between High Baseline Autophagy-Related ULK1 Expression and Improved Pain Control
Source: Arthritis. 2016 Jan 24;2016:4963950. doi: 10.1155/2016/4963950 (PMC4745296; doi:10.1155/2016/4963950)
Supplement: Supplementary file 1 — The autophagy marker ULK1 was significantly upregulated in a sample of RA patients (n = 16) at baseline compared to healthy subjects (Figure 1). Although there was a considerable variation in ULK1 gene expression, plotting the data without an outlier (n = 15) confirmed that RA patients exhibited significantly upregulated ULK1 gene expression (p = 0.01) at baseline (Supplementary Material, Figure 4). [file 4963950.f1.pdf]

SUPPLEMENTARY MATERIALS for the manuscript:

Rituximab downregulates gene expression in the peripheral blood from rheumatoid arthritis patients to levels found in healthy subjects: a link between high baseline autophagy-related ULK1 expression and improved pain control

by Elena V Tchetina et al.

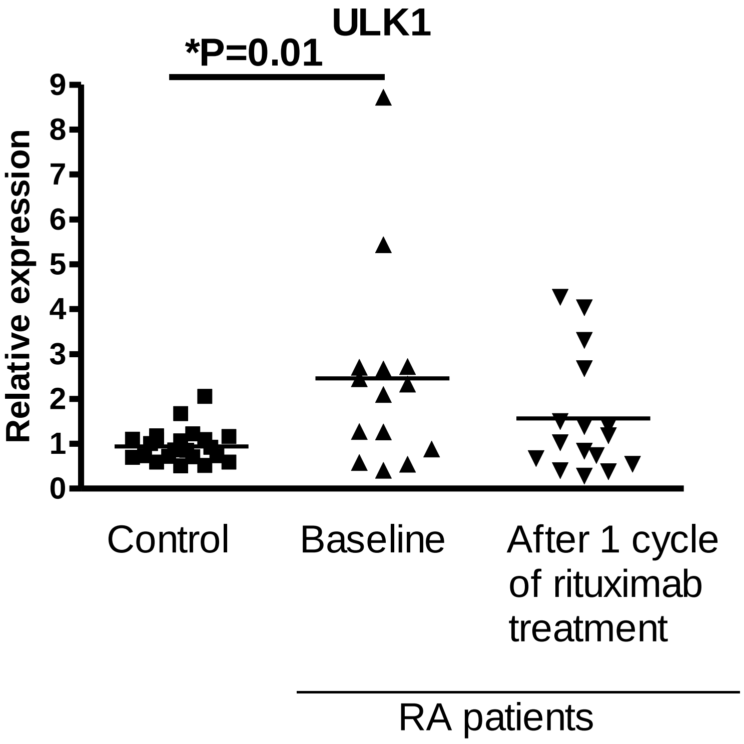

Figure 4. Relative expression of ULK1 without an outlier (n=1), with reference to β-actin as determined using real-time PCR analyses in whole blood from RA patients (n = 15) compared to healthy controls (n = 26). The controls are shown as 1.0 as required for relative quantification with the real-time PCR protocol. Asterisks (*) indicate significant differences from the control in pairwise comparisons (Mann-Whitney U-test). The number signs (#) show significant differences from baseline in pairwise comparisons (Wilcoxon matched pairs test).
